# Low zinc levels is associated with increased inflammatory activity but not with atherosclerosis, arteriosclerosis or endothelial dysfunction among the very elderly

**DOI:** 10.1016/j.bbacli.2014.07.002

**Published:** 2014-07-25

**Authors:** Rafaela C.S. De Paula, Ehimen C. Aneni, Ana Paula R. Costa, Valeria N. Figueiredo, Filipe A. Moura, Wladimir M. Freitas, Luiz A. Quaglia, Simone N. Santos, Alexandre A. Soares, Wilson Nadruz, Michael Blaha, Roger Blumenthal, Arthur Agatston, Khurram Nasir, Andrei C. Sposito, on behalf of the Brazilian Study on Healthy Aging Group

**Affiliations:** a State University of Campinas Medical School (UNICAMP), Campinas, SP, Brazil; b University of Brasilia Medical School (UnB), Brasilia, Brazil; c Johns Hopkins Ciccarone Center for the Prevention of Heart Disease, Baltimore, MD, USA; d University of Miami Miller School of Medicine, Miami, FL, USA; e Center for Prevention and Wellness, Baptist Health South Florida, Miami, FL, USA

**Keywords:** Zinc, Inflammation, Atherosclerosis, Arteriosclerosis, Endothelial dysfunction

## Abstract

**Background:**

Reduced zinc intake has been related to atherogenesis and arteriosclerosis. We verified this assumption in very old individuals, which are particularly prone to both zinc deficiency and structural and functional changes in the arterial wall.

**Methods:**

Subjects (n = 201, 80–102 years) with uneventful cardiovascular history and who were not in use of anti-inflammatory treatments in the last 30-days were enrolled. Daily intake of zinc, lipid profile, plasma C-reactive protein (CRP), plasma zinc, flow-mediated dilation (FMD), carotid ultrasonography and cardiac computed tomography were obtained. Young's Elastic Modulus, Stiffness Index and Artery Compliance were calculated.

**Results:**

There was no significant difference in clinical or laboratorial data between subjects grouped according to plasma zinc tertile, except for CRP (p = 0.01) and blood leukocytes (p = 0.002), of which levels were higher in the upper tertiles. The average daily intake of zinc was not significantly correlated with zinc or CRP plasma levels.

The plasma zinc/zinc intake ratio was inversely correlated with plasma CRP levels (− 0.18; p = 0.01). There was no significant difference between the plasma zinc tertiles and FMD, carotid intima–media thickness, coronary calcium score, carotid plaque presence, remodeled noncalcified coronary plaques, or low-attenuation noncalcified coronary plaques.

**Conclusion:**

Although plasma zinc level is inversely related to systemic inflammatory activity, its plasma levels of daily intake are not associated to alterations in structure or function of the arterial wall.

**General significance:**

In the very elderly plasma concentrations or daily intake of zinc is not related to endothelial dysfunction, arteriosclerosis or atherosclerotic burden at coronary or carotid arteries.

## Highlights

• Elderly are prone to nutritional deficiency, inflammation, and arterial wall diseases.
• Low zinc intake is related to artery wall diseases via stimulation of inflammatory activity.
• Plasma levels but not daily intake of zinc is related to systemic inflammatory activity.
• Plasma and dietary zinc are not associated to changes in arterial wall function or structure.

## 1 Introduction

In the very elderly, i.e. those aged 80 or more years, cardiovascular morbidity and mortality intensify due to a combination of prolonged exposure to traditional risk factors and rising of novel pro-atherogenic mechanisms [1]. At the core of this atherogenic process lies a persistent increase in systemic inflammation that is mainly attributed to immunosenescence, a reciprocal activation of the innate immune system due to an aging-dependent decline of the adaptive immune system. Recent data, however, have raised the possibility of reversible components that may contribute to this inflammatory upregulation in the elder.

Among potential candidates, zinc is a micronutrient that is essential for the immune system and its insufficiency may exacerbate immunosenescence. Indeed, individuals with inborn errors in zinc malabsorption present a reduced adaptive immunity that is compensated by the upregulation of innate immunity [2]. In the intracellular environment, zinc interacts with signal transducers implicated in immune response and influences both the structural stability and function of immunologically relevant transcription factors [3]. In the bloodstream, zinc insufficiency may also contribute to cardiovascular risk via its association with reduced anti-oxidant capacity [4], endothelial dysfunction [5], arterial wall stiffness and increased systolic blood pressure [6], [7].

The abovementioned arguments motivated the hypothesis that a higher zinc intake could help to mitigate a host of mechanisms that favor the decline of functional and structural properties of the arterial wall as well as the generation of atherosclerotic plaques. Since there is a particularly rapid decline in all these arterial properties among the very elderly, should zinc intake show to be beneficial, we expect its effect to be even more apparent in these individuals. So far, there is no information to confirm or refuse this hypothesis. Thus, the present study was designed to investigate the association pf zinc intake and plasma levels with arterial wall properties in a carefully selected cohort of very elderly individuals.

## 2 Subjects and methods

### 2.1 Participants

**Fig. 1:**
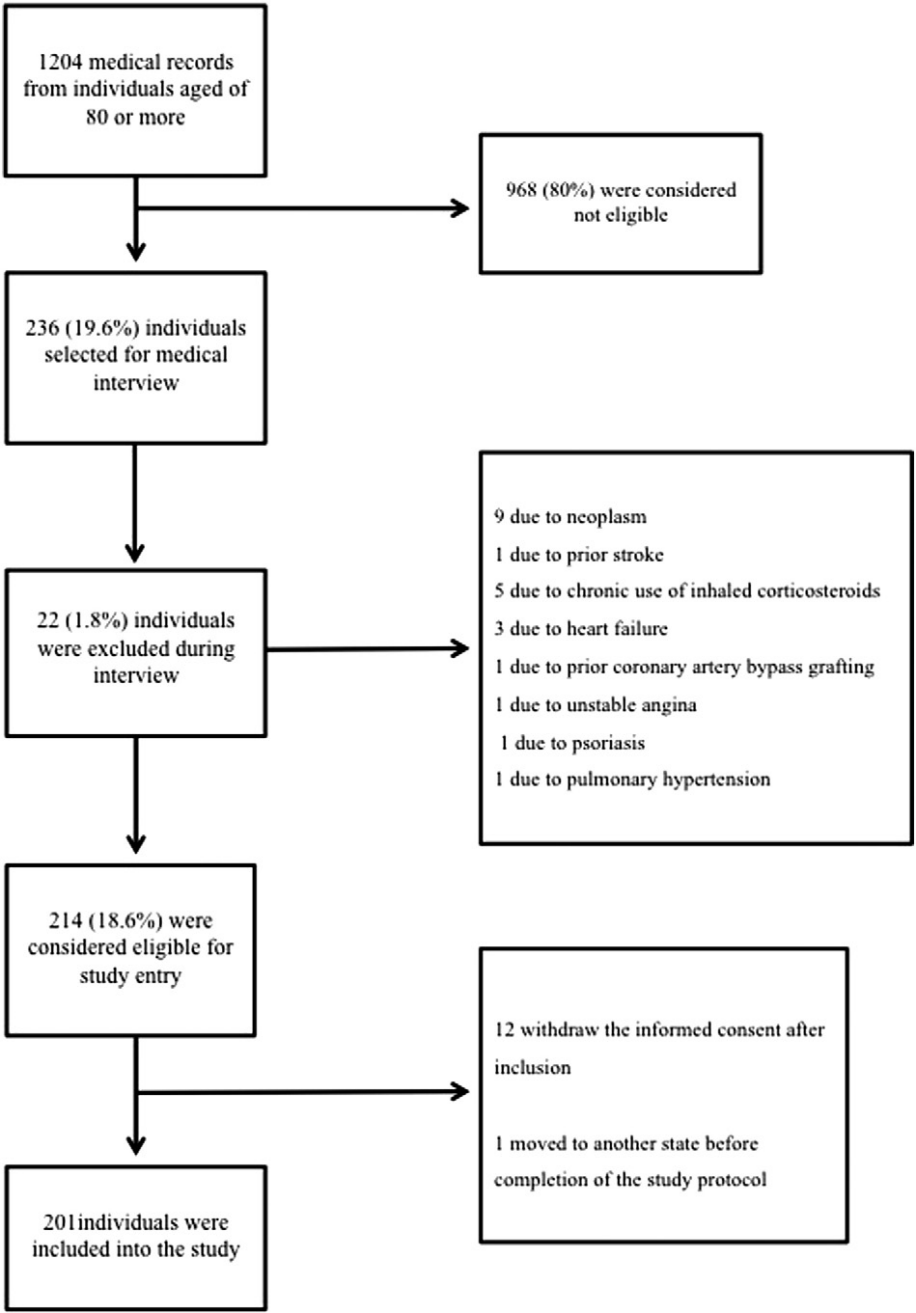
Flow diagram depicting the study design.

The studied population consisted of men and women who were consecutively recruited in the Brazilian Study on Healthy Aging from December 2008 to August 2011, as described elsewhere [8]. Briefly, after medical screening of 1204 individuals aged 80 years or more who spontaneously sought Biocardios Institute of Cardiology in Brasilia, Brazil, for assessment of cardiovascular risk, 214 (18.6%) were considered eligible, 12 (1%) chose not to participate, and 1 (0.08%) withdrew the informed consent due to impossibility to attend the exams and appointments. Thus, for the present investigation, 201 (16.7%) subjects were actually enrolled (Fig. 1). The main inclusion criterion was the absence of atherosclerotic coronary, cerebrovascular, and peripheral artery disease according to a medical evaluation, electrocardiogram, and echocardiogram. Exclusion criteria were: (i) functional dependency or institutionalization, (ii) abnormal cognitive ability as assessed by mini–mental state examination (< 13 points), (iii) use of anti-inflammatory treatments of any class in the last 30 days, (iv) current or previous diagnosis of cancer or immune inflammatory diseases, (v) chronic obstructive pulmonary disease, (vi) creatinine clearance < 25 ml/min/1.73 m^2^, (vii) significant hepatic disease (alanine aminotransferase or aspartate aminotransferase ≥ 1.5 the upper limit of normal), (viii) infectious disease manifested in the last 3 months, (ix) ejection fraction of the left ventricle < 50% on echocardiography, and (x) manifestation of neoplasia at admission or until the first year after enrollment. Screening for neoplasm was performed with the use of fecal occult blood test, mammography with clinical breast exam, Prostate-specific antigen plasma assay, digital rectal exam, and Papanicolaou smear according to current guidelines [9].

Study participants underwent blood collection for biochemical analysis, medical and nutritional evaluation, brachial artery reactivity, carotid ultrasonography, and cardiac computed tomography. All evaluations were performed in a time interval of up to one week. Smoking habit was defined for those who were currently smoking. Diabetes was defined as fasting glycemia ≥ 126 mg/dL or glycated hemoglobin (HbA1c) ≥ 6.5%. Metabolic syndrome was defined by the International Diabetes Federation criteria [10]. Physical inactivity was defined as less than 150 min of moderate intensity aerobic activity per week. Hypertension was defined if the systolic blood pressure (SBP) was ≥ 140 mm Hg or the diastolic blood pressure (DBP) was ≥ 90 mm Hg. The study was approved by the local ethics committee.

### 2.2 Biochemical analyses

Twelve-hour fasting plasma samples with EDTA were collected at admission and were immediately subjected to biochemical analyses in duplicates using an automatic chemical analyzer (Hitachi 917, Roche-Diagnostics). Glucose (Glucose GOD-PAP, Roche Diagnostics, Mannheim, USA), total cholesterol (CHOD-PAP, Roche Diagnostics, Mannheim, USA), triglycerides (GPO-PAP, Roche Diagnostics, Mannheim, USA), high-density lipoprotein cholesterol (HDL-C) (Roche Diagnostics, Mannheim, USA), high sensitivity C-reactive protein (CRP) (CardioPhase, Dade Behring, Marburg, USA), HbA1c (Variant II, Bio-Rad Laboratories, Hercules, CA, USA), apolipoprotein (apo) AI and B (Behring Nephelometer BNII, Dade Behring, Marburg, Germany) and plasma zinc (Atomic Absorption Spectrometry) were measured. Intra and interassay coefficients of variation for plasma zinc were 2.5% and 5.4%, respectively.

### 2.3 Assessment of dietary zinc intake and anthropometry

The body mass index (BMI) was calculated by the weight (in kilograms) divided by height (in meters) squared. Waist circumference was measured with the patient standing, using a non-stretch tape that circled the individual at the midpoint between the last rib and the iliac crest and the readings were taken at the time of expiration. Skinfolds were measured in triplicate, on the right side of the body, using a skinfold caliper (WCS Plus ®, Cardiomed, Curitiba, Brazil) to calculate the percentage of body fat using the sum of four skinfolds: biceps, triceps, subscapular and suprailiac.

A food frequency intake questionnaire (FFQ) previously validated in a Brazilian Population [11] was applied for assessment of zinc intake. Participants reported the intake of foods consumed during the previous month, which were clustered in 62 items, and the use of nutritional supplements. The middle portion in traditional measures of usual intake was standardized with the aid of a photographic record for dietary surveys and subsequently transformed into weight. Zinc content in vitamin supplements was considered the estimation of daily intake. The zinc intake was calculated based on food composition database of the Brazilian Table of Food Composition (TACO) [12]. Briefly, TACO is based on a systematic collection of samples of processed food, in triplicate, held in 9 cities of five geopolitical regions in Brazil (North, Northeast, South, Southeast and Midwest). The samples are composed of the main trademarks of the products (minimum of 3 and maximum of 5 for each product) and are collected in super/hypermarkets, which are responsible for about 85% of total food purchases in the country.

In order to validate the FFQ for estimating dietary intake of zinc, 21 study participants were evaluated by two board-certified nutritionists (RCSP and APR) in three different appointments within a maximum of one-week. Intra-observer and inter-observer variabilities were 1 ± 2% and 2 ± 2%, respectively. In addition, we also estimated the daily zinc intake of this subgroup of individuals by using a three-day weighed food record and a 24-hour diet recall. The estimated daily intake of zinc obtained by FFQ varied by 0.8 ± 3% as compared to 24-hour diet recall and by 4 ± 3% as compared with three-day weighed food record.

### 2.4 Carotid ultrasound

The intima–media thickness (IMT) and the presence of carotid plaques were assessed using high-resolution ultrasound (Philips, Model IE 33, 3–9 MHz transdutor linear, Philips Medical Systems, Andover, Massachusetts, USA) according to the protocols of the American Society of Echocardiography [13]. The carotid ultrasound bilateral measurements were made at the posterior wall of the common carotid artery and at the internal carotid artery through a program of automatic edge detection (QLAB version 6.0 software) to determine the mean and maximum IMT. Carotid plaque was defined as the presence of focal thickening of at least 50% higher than surrounding areas or as focal region with IMT > 1.5 mm and distinct adjacent edges. Bilateral end-diastolic and peak-systolic common carotid artery diameters were obtained by continuous tracing of 3 cycles and averaged to calculate, along with brachial blood pressure measurements, the following three indices of arterial elasticity: Young's Elastic Modulus (YEM), Stiffness Index (SI) and Artery Compliance (AC) [14]. Both YEM and SI give estimates of arterial stiffness and are considered relatively independent of wall thickness and blood pressure, respectively. AC measures the ability of the arteries to expand as a response to pulse pressure caused by cardiac contraction and relaxation.

### 2.5 Cardiac computed tomography

Cardiac CT was performed for analysis of coronary calcium score (CCS) on a 64-detector CT scanner (Aquillion 64, Toshiba, Ottawara/Japan). Axial slices of 3 mm thickness were acquired in synchrony with prospective electrocardiographic tracing in 70% of the RR interval. Coronary calcifications were measured and analyzed using the Agatston method. Contrast-enhanced CT angiograms were reconstructed from data acquisition windows with temporal resolution of 250 ms. The presence and characteristics of coronary atherosclerotic plaques were analyzed with the aid of a workstation (Vitrea ® — Vital Image) based on previously defined criteria [15]. Briefly, coronary arterial remodeling was defined when the diameter at the plaque site was at least 10% larger than the reference segment. Low-attenuation plaque was defined as non-calcified plaque with less than 30 HU.

### 2.6 Brachial artery reactivity

Exams were performed after 12-h overnight fasting and at least 24-h after withdrawal of vasoactive medications. After 10 min of rest in a quiet room with the temperature controlled around 22 °C, the brachial artery was located above the elbow, and a longitudinal image of 6 to 8 cm was taken as the resting scan. A blood pressure cuff was placed on the forearm and inflated to 50 mm Hg above the systolic blood pressure for 5 min. The cuff was deflated, and the flow-mediated dilation (FMD) scan was obtained during 2 min. The percent diameter change for FMD was calculated in relation to its respective rest scan. Brachial artery reactivity was analyzed by an experienced physician who was blinded to the patients' data. The intra-observer reproducibility was 95%.

### 2.7 Statistical methods

Normally distributed data are presented as mean ± SD and skewed data as median and interquartile range (IQR). Two-tailed ANOVA or Chi-square tests were used for comparison of the categorical baseline data. Analysis of covariance (ANCOVA) was used to assess the association between the magnitude of plasma zinc expressed as tertiles and the continuous normally distributed data. Adjustments for age and gender were performed in all comparisons across plasma zinc intervals. Assumptions of the ANCOVA models (linearity, normality of distribution and equal variance) were checked using histograms, normal probability plots, and residual scatter plots. Pearson test was used to examine correlation between plasma and dietary zinc levels. Multivariable ordinal logistic regression models were used to assess the relation between plasma zinc categories across increasing levels of plasma CRP levels with the reference group being patients in the lowest tertile. A two-sided p-value of 0.05 was considered statistically significant. All analyses were performed using IBM SPSS Statistics 20.0 for MAC (IBM, Armonk, NY, USA).

## 3 Results

Individuals enrolled into the study had a median level of schooling of 8(9) years, mini-mental exam score of 26(6). The average daily intake of zinc ranged from 3.3 to 52.2 mg or from 42 to 505% of the recommended daily intake (11 mg for men and 8 mg daily for women). Zinc containing supplement was reported by 31 participants. In these individuals, the total zinc intake was higher than in their counterparts (20.5 ± 10.1 vs. 8.8 ± 3.1 mg/day; p < 0.001). The use of zinc supplement increased the median zinc intake by 7.0 (0.5) mg/day. None of the individuals with zinc intake exceeding the recommended dietary allowance had plasma zinc levels below the minimum cutoff levels (70–72 μg/dL). From those who had less than the recommended daily intake, 5.4% had plasma zinc levels below the reference value. The average daily intake of zinc was not significantly correlated with zinc (p = 0.75) or CRP plasma levels (p = 0.76). Plasma zinc concentration was negatively correlated with plasma CRP (r = − 0.2; p = 0.003).

We calculated the ratio between plasma values and daily intake of zinc in order to infer the absorption of dietary zinc. The plasma zinc/zinc intake ratio increased significantly across increasing tertiles of plasma zinc (Table 1). This ratio was inversely correlated with plasma CRP levels (− 0.18; p = 0.01). The average daily zinc intake, however, remained uncorrelated with zinc plasma levels even after adjusting for gender, age, and plasma CRP levels (p = 0.5).

Clinical and laboratory characteristics of the enrolled patients are shown in Table 1, in which patients are segregated according to tertiles of plasma zinc levels. No differences were observed between the tertiles of plasma zinc, except for CPR (p = 0.01) and blood leukocytes (p = 0.002), of which levels were higher in the upper tertiles. We made a similar analysis comparing groups of individuals separated according to tertiles of daily intake of zinc. No significant difference was found between the groups in any of the tested variables (not shown).

In order to refine the association analysis adjusting for potential confounders, both plasma CRP and zinc levels were grouped into tertiles. Hence, multivariable ordinal logistic regression models were performed to assess the relation between CRP categories across different levels of plasma zinc while using the lowest CRP tertile as the reference group. In the unadjusted model (Model 1) and after progressive adjustment for age, gender, hypertension, diabetes, glomerular filtration rate, body fat percentage, and use of statins (Models 2 to 4), the highest tertile of zinc presented a direct association with higher CRP (Table 2). The average daily intake of zinc was not associated with CRP levels (p = 0.76) or with leukocytes (p = 0.68). We repeated the multivariable ordinal logistic regression models to assess the relation between CRP categories across different levels of daily intake of zinc. There was no significant association in unadjusted or adjusted models for this second regression analysis.

Also shown in Table 1, there was no significant difference in FMD, mean or maximal carotid IMT, frequency of IMT ≥ 1.0 mm, CAC, presence of carotid plaque, remodeled coronary plaque, or low-attenuation noncalcified coronary plaque between zinc tertiles. Likewise, there was no significant difference between the groups in carotid arterial compliance, stiffness index, or Young's modulus even after adjusting for age, gender, hypertension, diabetes, glomerular filtration rate, and use of statins (Table 1). These findings remained not statistically significant when we separated individuals based on their plasma zinc levels above or below the cutoff value for normality and compared the above-mentioned markers of arterial wall function and structure.

## 4 Discussion

The implication of plasma zinc in the pathogenesis of atherosclerotic and arteriosclerotic diseases has been raised due to its consistent association with systemic inflammatory markers. The present study verified this assumption in the very elderly, which is a population especially prone to selective nutritional deficiency, increased inflammatory activity, and arterial wall diseases. Although we found a significant association between systemic inflammatory activity and plasma zinc levels, there was no trace of a relation between plasma zinc and properties of arterial wall function or structure.

As commented above, recent studies demonstrated that low cell content of zinc is associated with increased secretion of inflammatory cytokines [16]. Although cellular and plasmatic concentrations of zinc are not comparable, an inverse relationship between plasma zinc and inflammatory activity has also been reported and was confirmed in our study. According to these immunomodulatory effects of zinc, one would expect that individuals presenting reduced plasma zinc levels could also have endothelial dysfunction and increased arterial wall stiffness and atherosclerotic burden. However, in our cohort, plasma zinc levels or daily intake of zinc was not associated with endothelial function, blood pressure, arterial wall stiffness, or atherosclerotic burden in carotid or coronary arteries. In a mechanistic point of view, as commented above, inflammatory activity affects zinc transporter activity in several tissues. This effect can change zinc distribution in plasma and tissues without affecting the total zinc store. By inference, it is likely that in individuals with mildly elevated systemic inflammatory activity, such as that induced by atherosclerosis or aging, plasma zinc levels do not reflect tissue zinc status, but are rather indicative of the acute phase inflammatory response. This assumption must be confirmed in further investigations.

Such lack of association between zinc intake/plasma concentration and cardiovascular disease was similarly reported in younger adults [17], [18] and in the prospective Physicians' Health Study II [19]. In this latter study, the same incidence of cardiovascular events was found in individuals aged 50 years or older that were randomized to nutritional supplementation with the daily-recommended dose of zinc (11 mg/day) or placebo during 11.2 years. Thus, our current findings correspond to previous evidences and suggest that the lack of association is extended to the very elderly. This may be particularly concerning considering that Americans spend around $5.2 billion a year on multivitamin/mineral supplements [20] and by the age of 71 years or older, 36% are taking zinc supplements [21]. So far, evidence remains unsupportive of this expenditure for cardiovascular disease prevention.

The association between systemic inflammation and plasma zinc concentration was found both in individuals who had half and those who had up to five times the recommended dietary allowance of zinc [22], indicating a link that is regardless of zinc intake. Consistently, in patients with rheumatoid arthritis, disease activity is a better predictor of plasma zinc than dietary factors [23]. In this context, we found that the ratio between the plasma values and dietary intake of zinc was inversely correlated with CRP plasma levels, suggesting that zinc absorption and systemic inflammation are linked. Indeed, increased inflammatory activity was previously found to be predictive of reduced zinc transporter activity in individuals with type 2 diabetes mellitus [24] and obesity [25].

Besides the effect of inflammation of zinc transporters and, by consequence, its absorption from diet, plasma zinc concentration is also sensitive to its distribution between extracellular fluid pool and tissues. In an animal model, it was shown that the liver expression of zinc transporters is upregulated by interleukin-6 [26]. Conceivably, since interleukin-6 is also a major stimulus for CRP synthesis by the liver, an enhanced production of this cytokine, as observed in aged individuals, may simultaneously underlie both the production of CRP and the increase of hepatic uptake of zinc. Thus, although in healthy individuals the dietary zinc intake is related to both its store and plasma concentration, [27] this association declines due to the increased inflammatory activity observed among the elderly [1].

Some limitations must be borne in mind when interpreting the present findings. The first and most important limitation resides in the external validity of our findings. We aimed to investigate cardiovascular risk markers among elderly in a primary prevention setting and to avoid some potential confounders we excluded individuals with neoplastic diseases, malnutrition, or other conditions that may influence the immunoinflammatory response. Consequently, we achieved a high exclusion rate (83%). Another important limitation is the lack of data concerning zinc transporter activity and zinc intracellular content. So far, these measurements are still hard to be obtained in clinical studies and are mostly limited to indirect assessment via measuring messenger RNA levels from leukocytes. Thus, future studies and new assessment methods are required to better clarify this complex association between zinc homeostasis and cardiovascular disease. Finally, while the sample size was enough for detecting CRP differences it was insufficient to exclude small differences in artery wall functional or structural parameters between groups. However, considering the difference we found in the mean or max IMT between tertiles it would be necessary to enroll as much as 50,000 individuals to obtain a satisfactory statistical power (ß = 0.8) to exclude this difference. Even though this difference would become significant after such an enlargement of sample size, its relevance would remain questionable based on this large variation and small differences of the means.

In conclusion, this study showed a negative and independent association between plasma zinc levels and inflammatory activity in a population of very elderly individuals. This association, however, did not have any impact on endothelial function, arteriosclerosis, or atherosclerotic burden at carotid or coronary arteries.

## Conflict of interest

The authors declare that they have no conflicts of interest.

## Figures and Tables

**Table 1:** Characteristics of the patients according to their plasma zinc concentrations.

Characteristics	1st tertile < 92 μg/dL	2nd tertile 92–107 μg/dL	3rd tertile > 107 μg/dL	p
Plasma zinc, μg/dL	81 ± 10	100 ± 4	120 ± 16	
Sample size	69	66	66	
Men, n[%]	23[33]	27[41]	15[23]	0.25
Age, years	85 ± 5	84 ± 3	84 ± 4	0.06
BMI, kg/m^2^	27 ± 6	25 ± 4	26 ± 4	0.18
Body fat, %	35 ± 6	37 ± 6	36 ± 7	0.06
SBP, mm Hg	144 ± 20	147 ± 22	144 ± 19	0.79
DBP, mm Hg	75 ± 11	76 ± 11	74 ± 11	0.12
HR, bpm	76 ± 12	73 ± 12	71 ± 10	0.08
Waist circumference, cm	93 ± 13	91 ± 13	94 ± 10	0.23
Daily intake of zinc, mg	11.3 ± 6.8	12.0 ± 9.4	10.3 ± 5.6	0.75
Plasma zinc/zinc intake ratio	8.9 ± 3.9	11.7 ± 6.1	14.1 ± 6.4	0.001
Hypertension, n[%]	51[77]	46[69]	56[81]	0.22
Time from hypertension diagnosis, years	21(18)	26(21)	21(23)	0.60
Smoking, n[%]	0	4[2.5]	1[1.5]	0.10
Physical inactivity, n[%]	52[82]	43[67]	47[70]	0.11
Diabetes, n[%]	19[29]	15[22]	13[19]	0.34
Time from diabetes diagnosis, years	9(19)	7(10)	12(30)	0.40
Metabolic syndrome, n[%]	39[59]	33[50]	39[57]	0.55
Prior statin use, %	46	33	35	0.21
Glycated hemoglobin, %	6.4 ± 1.6	6.0 ± 0.6	6.2 ± 1.0	0.18
Glucose, mg/dL	110 ± 50	99 ± 20	103 ± 43	0.25
Total cholesterol, mg/dL	196 ± 42	201 ± 41	198 ± 40	0.78
HDL cholesterol, mg/dL	55 ± 14	53 ± 14	56 ± 13	0.55
LDL cholesterol, mg/dL	110 ± 36	117 ± 38	115 ± 36	0.54
Triglycerides, mg/dL	125 ± 51	136 ± 66	121 ± 50	0.27
Apo A, mg/dL	149 ± 27	148 ± 23	155 ± 33	0.22
Apo B, mg/dL	85 ± 23	91 ± 28	83 ± 23	0.20
CRP, mg/L	2.0(2.0)	1.7(2.0)	1.4(1.5)	0.01
Leucocytes, cells/mm^3^	6562 ± 1499	6213 ± 1472	6127 ± 1706	0.002
Mean IMT, mm	0.83 ± 0.11	0.86 ± 0.13	0.83 ± 0.13	0.36
Mean max IMT, mm	1.6 ± 0.6	1.6 ± 0.6	1.7 ± 0.6	0.36
Individuals with IMT ≥ 1 mm, n[%]	8[40]	14[70]	9[45]	0.78
Individuals with carotid plaque, n[%]	14[70]	14[70]	18[82]	0.60
CAC, Agatston	166(361)	176(461)	114(414)	0.20
Individuals with remodeled coronary plaque, n[%]	22[44]	26[47]	24[44]	0.91
Individuals with low-attenuation, noncalcified coronary plaque, n[%]	25[50]	30[55]	27[49]	0.83
Carotid arterial compliance, %/10 mmHg	0.74(0.70)	0.82(0.70)	0.89(0.80)	0.34
Carotid Stiffness index	18(31)	11(15)	16(21)	0.53
Carotid Young's modulus, mm Hg·mm	1262(2453)	1048(1222)	1197(1668)	0.44
FMD, %	3.4(5.9)	6.1(6.7)	4.3(5.6)	0.26

Values are expressed as percentage or mean ± standard deviation or median [interquartile range]. Apo: apolipoprotein; BMI: body mass index, SBP: systolic blood pressure, DBP: diastolic blood pressure, HR: heart rate; IMT: carotid artery intimal medial wall thickness; CAC: coronary artery calcification; FMD: flow-mediated dilation; HDL: high-density lipoprotein, LDL: low-density lipoprotein, CRP: C-reactive protein.

**Table 2:** Ordinal regression analysis according to tertiles of levels of C-reactive protein.

	1st tertile (≤ 1.1 mg/L)	2nd tertile (1.2 to 2.4 mg/L)	3rd tertile (> 2.4 mg/L)
Sample size	60	61	64
Plasma-zinc levels	< 92 μg/dL	92–107 μg/dL	> 107 μg/dL
Model 1	Ref group	0.72 (0.37–1.40)p = 0.335	0.45 (0.23–0.87)p = 0.018
Model 2	Ref group	0.83 (0.42–1.64)p = 0.59	0.50 (0.25–0.97)p = 0.041
Model 3	Ref group	0.84 (0.42–1.67)p = 0.61	0.46 (0.23–0.90)p = 0.024
Model 4	Ref group	0.88 (0.44–1.76)p = 0.71	0.50 (0.25–0.99)p = 0.049

Ref = reference. Model 1 is unadjusted; Model 2 is adjusted for age and gender; Model 3 is adjusted for age, gender, hypertension, diabetes, glomerular filtration rate and plasma apolipoprotein-B; Model 4 is adjusted for age, gender, hypertension, diabetes, glomerular filtration rate and use of statins.
